# High Expression of Claudin-4 Is Associated with Synchronous Tumors in Patients with Early Gastric Cancer

**DOI:** 10.3390/jcm11123550

**Published:** 2022-06-20

**Authors:** Won Shik Kim, Hayeon Kim, Moon Kyung Joo, Byung Il Choi, Ah Young Yoo, Jong-Jae Park, Beom Jae Lee, Seung Han Kim, Hoon Jai Chun

**Affiliations:** 1 Division of Gastroenterology, Department of Internal Medicine, Korea University Guro Hospital, Korea University College of Medicine, 148, Gurodong-ro, Guro-gu, Seoul 08308, Korea; ws907568@gmail.com (W.S.K.); bichoi86@hanmail.net (B.I.C.); person88@naver.com (A.Y.Y.); gi7pjj@korea.ac.kr (J.-J.P.); l85210@korea.ac.kr (B.J.L.); kimseunghan09@gmail.com (S.H.K.); 2 Department of Pathology, Korea University Guro Hospital, Korea University College of Medicine, 148, Gurodong-ro, Guro-gu, Seoul 08308, Korea; kimhayeon223@korea.ac.kr; 3 Division of Gastroenterology, Department of Internal Medicine, Korea University Anam Hospital, Korea University College of Medicine, 73, Inchon-ro, Seongbuk-gu, Seoul 02841, Korea; drchunhj@gmail.com

**Keywords:** claudin-4, early gastric cancer, synchronous neoplasms

## Abstract

Claudin (CLDN) is a tight junction protein found in human epithelial cells and its altered expression is known to be associated with the progression of gastric cancer. We aimed to investigate the differential expression of CLDN-4 in early gastric cancer (EGC) according to its clinicopathological characteristics. We enrolled 53 patients with EGC who underwent surgical gastric resection from January 2007 to December 2018. The staining intensity of the tumor cells was scored as 0–3, and the percentage of staining was scored as 0–5; high expression was defined if the intensity plus percentage score was 7 or 8, and low expression was defined if the score was 0–6. Among the 53 patients, 16 (30.2%) showed low CLDN-4 expression, while 37 (69.8%) had high CLDN-4 expression. High CLDN-4 expression was significantly associated with intestinal-type EGC (low: 12.5% vs. high: 56.8%, *p* = 0.003), open-type atrophic change (low: 60.0% vs. high: 90.9%, *p* = 0.011), and the presence of synchronous tumors (0 vs. 32.4%, *p* = 0.010), and all 12 EGCs with synchronous tumors showed high CLDN-4 expression. However, expression of CLDN-3, a typical intestinal phenotype CLDN, was neither correlated with CLDN-4 expression nor associated with synchronous tumors. Taken together, high CLDN-4 expression may be considered as an auxiliary tool for screening synchronous tumors in patients with EGC.

## 1. Introduction

Epithelial and endothelial cells provide a protective barrier in multiple organs and aid in the maintenance of homeostasis. Protective barriers include tight junctions (TJs), adherens junctions, and desmosomes. The TJ barrier plays a major role in maintaining the intercellular space of the epidermal granular cell layer and controls intercellular communication and paracellular transport. In the stomach, TJs act as barriers to prevent the movement of substances, such as water, ions, and protein molecules, through the paracellular pathway, while simultaneously dividing the cell membrane into apical and basolateral regions to maintain cell polarity [1]. Tight junction-associated proteins, such as occludin, claudin (CLDN), and junctional adhesion molecules, are involved in the TJ structure [2,3].

As more studies on cancer growth and metastasis have been conducted, interest in TJs has increased, and several studies have reported that the TJ plays an important role in cancer progression [4,5]. CLDN, a transmembrane protein with a size of approximately 20–27 kDa, promotes cell–cell adhesion by acting as a constituent of TJs. CLDN passes through the cell membrane four times, with both N- and C-termini located in the cytoplasm. TJs not only block the entry of foreign substances or ions by completely blocking the cell–cell junction, but also maintain cell polarity, and are involved in other functions, such as cell proliferation and differentiation by interacting with signaling proteins. In addition, CLDN is involved in the transformation into a malignant tumor through a phenomenon called epithelial–mesenchymal transition (EMT). CLDN subtypes 1–4 were found to be widely expressed in all human tissues [6]. CLDN-4 has further been identified as one of the markers of gastric adenocarcinoma precursor lesions [7,8] and found to be expressed in gastric adenocarcinoma [9]. However, it remains unclear whether CLDN-4 is related to the progression and outcome of gastric carcinoma [8,10].

With the development of endoscopic technology, gastric tumors can be more easily detected using image-enhanced endoscopy, such as narrow band imaging (NBI), and the number of missed lesions has been decreasing. However, multiple tumors may exist in the chronic inflammatory background mucosa and synchronous tumors may not be detected. According to previous reports, the incidence of synchronous multiple gastric cancer is more common (3.2–12.3%) in early gastric cancer patients [11,12,13,14,15] than in those with advanced gastric cancer [16,17]. Endoscopists need to pay attention to novel methods to easily detect multiple synchronous lesions; however, such methods have not been clearly established yet. Therefore, we investigated the differential expression of CLDN-4 in EGC according to various clinicopathological characteristics, focusing especially on the presence or absence of synchronous tumors.

## 2. Materials and Methods

### 2.1. Study Populations and Tissue Preparation

This study was conducted on 53 patients diagnosed with EGC who underwent surgical resection using specimens collected from the bank of human-derived materials at Korea University Guro Hospital, from January 2007 to December 2018. The patients in this study were recruited based on the quality of the blocks and the presence of complete clinical records. Complete clinical data, such as age, gender, and clinical picture, were obtained from the patients’ clinical records. The study protocol was reviewed and approved by the Institutional Review Board of Korea University Guro Hospital (IRB number 2019GR0449). Due to the retrospective nature of the study, the requirement of informed consent was waived by the board.

### 2.2. CLDN Expression Immunocytochemistry

CLDN expression was determined by immunohistochemical staining using the avidin–biotin complex immunoperoxidase method. The tissues obtained from primary cancer tissue (5 μm slides) were deparaffinized in xylene (Abcam, Cambridge, MA, USA) and rehydrated in a graded series of ethanol (Abcam, Cambridge, MA, USA), followed by microwave antigen retrieval (DAKO Real Target Retrieval Solution 10x #S2031. Agilent Scientific Instruments., Santa Clara, CA, USA). The sections were incubated overnight (12 h) at 4 °C with human anti-claudin-3 (ab15102, 1:200) and anti-claudin-4 (ab 210736, 1:500) (Abcam, Cambridge, MA, USA) primary antibodies. Immunohistochemical staining was conducted using donkey anti-mouse IgG (H+L)-Alexa488 (1:500; Jackson ImmunoResearch, West Grove, PA, USA) and 1 μg/mL of DAPI (Invitrogen, Waltham, MA, USA). The sections were counterstained with Meyer’s hematoxylin (Abcam, ab220365, Cambridge, MA, USA), dehydrated, cleared, and mounted. Normal gastric mucosa samples were used as positive controls.

**Figure 1:**
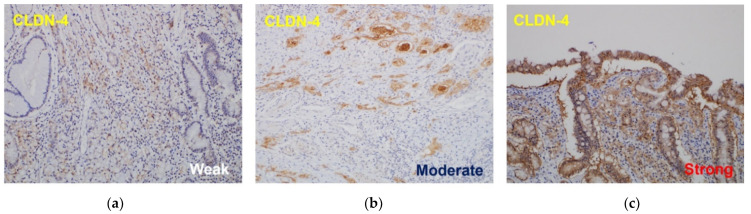
The results of claudin-4 expression in immunohistochemical staining: (**a**) weak expression of CLDN-4, (**b**) moderate expression of CLDN-4, (**c**) strong expression of CLDN-4. CLDN, claudin.

The staining intensity score ranged from 0 to 3 (0: negative, 1: weak [weak or fragmented membranous pattern], 2: moderate [moderately intense membranous staining], and 3: strong [strong, well-localized, linear circumferential membranous staining pattern]), and the percentage score ranged from 0 to 5 (0: none, 1: <1/100, 2: 1/100 to 1/10, 3: 1/10 to 1/3, 4: 1/3 to 2/3, 5: >2/3). The intensity and percentage scores were added, and 0 to 6 points were defined as low expression, and 7 to 8 points were defined as high expression [18] (Figure 1).

### 2.3. Definitions

All patients underwent endoscopic inspection within 1 month before surgery. The pre-operational extent of atrophic gastritis was classified as closed-type or open-type according to the Kimura and Takemoto classification [19]. A synchronous tumor was defined as a separate dysplastic or cancerous tumor in the surgical specimen or tumors detected by endoscopic examination within 6 months of the first diagnosis of index EGC [20]. Synchronous multiple tumors were defined as two or more neoplastic lesions detected simultaneously in a pathological examination after the diagnosis of index EGC.

### 2.4. Statistical Analyses

Continuous variables were expressed as mean ± standard deviation. The χ^2^ test or Student’s *t*-test for independent samples was performed to assess differences in risk factors between the groups. The results were considered statistically significant if *p* < 0.05. All statistical analyses were performed using the Statistical Package for the Social Sciences software (version 20.0; IBM Corp., Armonk, NY, USA).

## 3. Results

### 3.1. Clinical and Histopathologic Features of the CLDN-3 and CLDN-4 Low- and High-Expression Groups

A total of 53 patients were enrolled in this study; the male:female ratio was 31:22, and their mean age was 64.1 ± 11.7 years. Among the 53 patients, 16 (30.2%) were classified into the CLDN-4 low-expression group, and 37 (69.8%) were classified into the high-expression group. Compared with the CLDN-4 low-expression group, patients in the CLDN-4 high-expression group were significantly older (59.2 vs. 66.9 years, *p* = 0.031). Interestingly, all 12 patients who had synchronous tumors showed high expressions of CLDN-4, while none of the patients in the low-expression group had synchronous tumors (0 vs. 32.4%, *p* = 0.010). The sensitivity, specificity, positive predictive value, and negative predictive value of high CLDN-4 expression for synchronous tumors were 100, 26.4, 22.6%, and 100%, respectively (area under the curve = 0.695, Supplementary Figure S1). However, the male-to-female ratio, tumor location, and tumor size were not significantly different between the two groups. As CLDN-3 is known to have a typical intestinal phenotype like CLDN-4, we also investigated the difference of various characteristics according to the CLDN-3 expression level. In contrast to CLDN-4, the CLDN-3 high-expression group was frequently located at the lower third compared to the CLDN-3 low-expression group (73.4% vs. 31.6%, *p* = 0.006); however, other variables such as gender, age, tumor size, and the presence of synchronous tumor were not significantly different between high and low expressions of CLDN-3. The baseline characteristics of the enrolled patients are shown in Table 1.

In terms of histopathological features, the CLDN-4 high-expression group showed a high frequency of the differentiated histology (51.4% vs. 18.8%, *p* = 0.027) and intestinal type (56.8% vs. 12.5%, *p* = 0.003). However, other features, such as tumor depth, lymphovascular or lymph node invasion, venous or perineural invasion, and high microsatellite instability (MSI), were not significantly different between the two groups. Furthermore, all histopathologic features were not significantly different between the high and low expressions of CLDN-3. The histopathological features of the CLDN-4 low- and high-expression groups are presented in Table 2.

### 3.2. Association between Atrophic Range, CLDN-3, and CLDN-4 Expression

**Figure 2:**
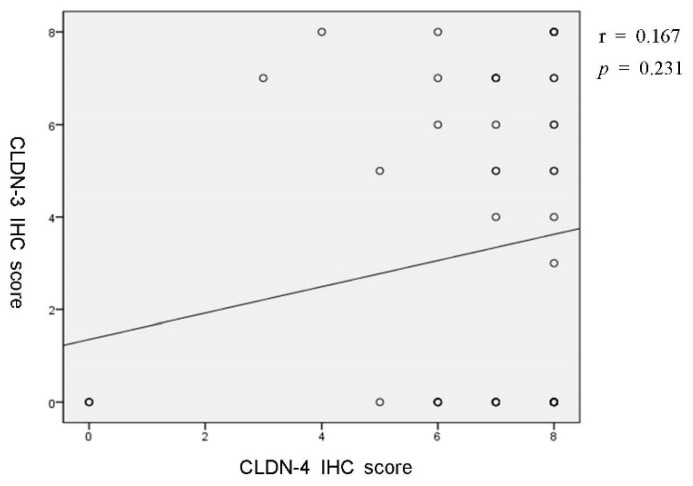
Correlation between CLDN-3 IHC score and CLDN-4 IHC score. CLDN, claudin; IHC, immunohistochemistry.

As we found that the CLDN-4 high-expression group showed more differentiated-type histology and intestinal-type EGC, we hypothesized that high expressions of CLDN-4 could be linked to a wide range of precancerous lesions, such as atrophic gastritis, which may contribute to the differentiated-type histology, intestinal-type EGC, and predominant synchronous tumors. Thus, we further investigated the relationship between CLDN-4 expression and a range of atrophy. As expected, 90.9% of open-type atrophy was observed in the CLDN-4 high-expression group, whereas only 60.0% of the low-expression group showed the open type (*p* = 0.011). However, high expressions of CLDN-3 were not associated with open-type atrophy, which was not consistent with the results from CLDN-4 (*p* = 0.760) (Table 3). Finally, we investigated the correlation between CLDN-3 and CLDN-4 expression levels. Among the 53 patients, 38 (71.1%) had low expression and 15 (28.3%) showed high expression of CLDN-3. However, low and high expressions of CLDN-3 were not significantly correlated with low and high CLDN-4 expression (r = 0.167, *p* = 0.231, Figure 2). Taken together, these findings suggest that the predictive value for synchronous tumors among EGC patients may be confined to CLDN-4, not CLDN-3.

## 4. Discussion

In this study, we found that a high expression of CLDN-4 in EGC was significantly associated with old age, differentiated histology and intestinal-type EGC, open-type atrophy, and the presence of synchronous tumors. The sensitivity and negative predictive value of high CLDN-4 expression in synchronous tumors were 100% in our study. Thus, we suggest that high CLDN-4 expression in EGC tissues may be useful for predicting synchronous tumors in other areas of the stomach. This information could be helpful for endoscopists, who should pay attention to the detection of multiple dysplastic or cancerous lesions in the stomach, especially in patients with a previous history of gastric cancer.

Several reports have described missed synchronous gastric cancer after endoscopic submucosal dissection (ESD) or surgical gastrectomy [21,22]. One study reported that more than half of cases with missed synchronous lesions had images of missed lesions captured during the previous endoscopy examination [23]. The delayed detection of synchronous lesions may lead to additional treatment including ESD or surgery that can result in increased patient discomfort and medical costs. The early detection of synchronous gastric lesions before progression to invasive cancers is an important issue for improving patient prognosis. Previous studies have shown that the expression level of CLDN-4 is higher in intestinal metaplasia (IM) and dysplasia than in the normal mucosa [7,8,24]. Our findings support the value of high CLDN-4 expression as an auxiliary marker for the prediction of synchronous tumors as well as early cancer generation.

In previous studies, older age, male gender, smaller tumor size, well-differentiated type, less deep invasion, atrophic gastritis, and IM were reported to be associated with synchronous multiple EGCs [14,15,25,26]. A previous retrospective study in Japan evaluated the degree of atrophy in endoscopy and the risk of gastric cancer. In the case of severe atrophy, the annual incidence of gastric cancer was 0.31%, which was about three times higher than that in the case of less atrophic gastritis [27]. Another study also reported that the cumulative incidence of gastric cancer was highly associated with the extent of atrophic gastritis [28]. Furthermore, the consistent cancerogenic background may result in the development of two or more lesions with similar clinicopathological characteristics. Considering these factors, precancerous lesions such as atrophic gastritis and IM could offer a dysplastic area where gastric cancer may arise simultaneously at different sites [29]. In our study, more than 90% of the highly expressed CLDN-4 group showed open-type atrophy, and we considered that high expression of CLDN-4 in EGC may be linked with severe atrophic gastritis and the subsequent presence of synchronous tumors.

However, it is not yet clear how the high expression of CLDN-4 is related to the occurrence of synchronous lesions. In the stomach, CLDNs are classified into the gastric (i.e., CLDN-18) and intestinal (i.e., CLDN-3 and CLDN-4) phenotypes. Multiple pathways, including the signal transducer and activator of transcription 3 (STAT3), *Helicobacter pylori* (*H. pylori*), the PI3K/Akt pathway, toll-like receptor 2 (TLR2), caudal-type homeobox 2 (Cdx2), Snail, E-cadherin, MMP, and CpG island hypermethylation, are all associated with CLDN-4 expression in precancerous GC lesions [30]. In particular, Cdx2, the caudal-related transcription factor, affects the regulation of intestinal CLDN expression both in vivo and in vitro. The expression of Cdx2 is known to be associated with intestinal CLDN proteins, suggesting an important role in the regulation of intestinal CLDN in GC, as well as in IM [31]. In addition, Cdx2 can induce IM and regulate the expression of CLDN-4 during the intestinal differentiation of GC [32]. Thus, we consider that Cdx2 may act as a molecular link between CLDN-4 expression and the presence of precancerous lesions and synchronous tumors.

Because the expression patterns of CLDNs are diverse and dynamic, the transcription and function of CLDNs must be tightly controlled via a wide range of regulatory mechanisms. Among these mechanisms, EMT plays an important role [33]. The malignant progression of many types of carcinomas, possibly all of them, depends on the EMT activation of neoplastic cells [34,35,36]. The transition to a more mesenchymal-like phenotype may promote the endovascular invasion of tumor cells from surrounding vessels and migration to new organs. In pathogenesis of cancer, neoplastic cells during the early stage of carcinoma are in an epithelial-like state, gradually acquiring more mesenchymal characteristics as the tumor progresses. CLDN-4 is expressed on the cell membranes of various tissues and is most abundantly expressed in the gastrointestinal tract [37]. Since the loss of cell–cell adhesion complexes is associated with increased EMT in cancer, the reduction in CLDN activity may promote metastasis and invasion. Alternatively, the overexpression of CLDNs has also been reported to increase aberrant localization and function in gastric, lung, prostate, ovarian, colorectal, and breast cancers, promoting metastasis and progression [38]. In our study, low or high CLDN-4 expression was not significantly associated with the tumor invasion depth, or lymphovascular, nodal, venous, or perineural invasion. Due to the limited number of enrolled patients, we believe that it is premature to state the role of CLDN-4 in the progression of EGC.

This study had several limitations. First, this study comprised a retrospective cohort analysis with a small sample size from a single center and only included patients who underwent surgical resection. This stringent selection could introduce selection bias, and the generalizability of the results is low. Second, not all patients in this cohort underwent total gastrectomy; this raises the possibility of missing lesions, which may underestimate the incidence of concurrent multiple EGCs. However, all patients underwent surveillance endoscopy within 6 months, and synchronous tumors of remnant stomach among patients who underwent subtotal gastrectomy could be detected using endoscopic surveillance. Third, several other risk factors, such as *H. pylori* infection, a family history of gastric cancer, smoking status, dietary patterns, and other genetic mutation markers (e.g., p53 and CDH1) were not studied. The consistent value of CLDN-4 after adjusting for these clinicopathological factors should be further analyzed in future studies. Fourth, we could not validate the value of CLDN-4 for the prediction of synchronous tumors of EGC patients in an independent cohort. Further analysis such as the validation of the role of CLDN-4 using an open database set of genetic profiles of gastric cancer patients or the development of a machine learning system including information on CLDN-4 expression for the prediction of synchronous tumors should be performed in the future.

## 5. Conclusions

We demonstrated that high CLDN-4 expression is a potential predictor of synchronous neoplasms. Although further clinical studies are required to support these findings, CLDN-4 shows potential as an auxiliary tool for screening synchronous tumors in patients with EGC.

## Figures and Tables

**Table 1:** Baseline clinical characteristics according to high and low expressions of CLDN-3 and CLDN-4.

Variables	Total (*n* = 53)	CLDN-3 Low (*n* = 38)	CLDN-3 High (*n* = 15)	*p*-Value	CLDN-4 Low (*n* = 16)	CLDN-4 High (*n* = 37)	*p*-Value
Male:female (*n*)	31:22	24:14	7:8	0.272	7:9	24:13	0.152
Age (age, ±SD)	64.1 ± 11.7	64.2 ± 11.3	65.5 ± 13.0	0.183	59.2 ± 11.2	66.9 ± 11.2	0.031
Tumor location (%, *n*)				0.018			0.397
Upper third	7.5 (4)	10.5 (4)	0 (0)	0.191	0 (0)	10.8 (4)	0.171
Middle third	49.1 (26)	57.9 (22)	26.7 (4)	0.041	62.5 (10)	43.2 (16)	0.198
Lower third	43.4 (23)	31.6 (12)	73.4 (11)	0.006	37.5 (6)	49.1 (17)	0.569
Tumor size (cm, ±SD)	4.0 ± 2.2	3.8 ± 2.2	4.7 ± 2.3	0.183	3.4 ± 1.8	4.4 ± 2.4	0.111
Synchronous tumor (%, *n*)	22.6 (12)	21.1 (8)	26.7 (4)	0.660	0 (0)	32.4 (12)	0.010

CLDN, claudin; SD, standard deviation.

**Table 2:** Histopathologic features according to high and low expressions of CLDN-3 and CLDN-4.

Variables	Total (*n* = 53)	CLDN-3 Low (*n* = 38)	CLDN-3 High (*n* = 15)	*p*-Value	CLDN-4 Low (*n* = 16)	CLDN-4 High (*n* = 37)	*p*-Value
Differentiation (%, *n*)				0.272			0.027
Differentiated type	41.5 (22)	36.8 (14)	53.3 (8)	0.272	18.8 (3)	51.4 (19)	0.027
Undifferentiated type	58.5 (31)	63.2 (24)	46.7 (7)	0.272	81.2 (13)	48.6 (18)	0.027
Lauren classification (%, *n*)				0.933			0.008
Intestinal	43.4 (23)	44.7 (17)	40.0 (6)	0.754	12.5 (2)	56.8 (21)	0.003
Diffuse	45.3 (24)	44.7 (17)	46.7 (7)	0.899	75.0 (12)	32.4 (12)	0.004
Mixed	11.3 (6)	4 (10.5)	13.3 (2)	0.771	12.5 (2)	10.8 (4)	0.859
SM invasion (%, *n*)				0.650			0.066
SM1	43.4 (23)	47.4 (18)	33.3 (5)	0.353	56.2 (9)	37.8 (14)	0.214
SM2	22.6 (12)	21.1 (8)	26.7 (4)	0.660	25.0 (4)	21.6 (8)	0.787
SM3	34.0 (18)	31.6 (12)	40.0 (6)	0.560	18.8 (3)	40.5 (15)	0.124
LV invasion (%, *n*)	56.6 (30)	50.0 (19)	73.3 (11)	0.123	43.8 (7)	62.2 (23)	0.213
LN positivity (%, *n*)	43.4 (23)	42.1 (16)	46.7 (7)	0.763	43.8 (7)	43.2 (16)	0.973
Venous invasion (%, *n*)	0 (0)	0 (0)	0 (0)	1.000	0 (0)	0 (0)	1.000
Perineural invasion (%, *n*)	1.9 (1)	2.6 (1)	0 (0)	0.526	0 (0)	2.7 (1)	0.507
MSI high * (%, *n*)	12.2 (6)	11.4 (4)	14.3 (2)	0.783	20.0 (3/15)	8.8 (3/34)	0.271

CLDN, claudin; SM, submucosa; LV, lymphovascular; LN, lymph node; MSI, microsatellite instability.* Examined in 49 patients.

**Table 3:** Atrophy type according to high and low expressions of CLDN-3 and CLDN-4.

Variables	CLDN-3 Low (*n* = 34) *	CLDN-3 High (*n* = 14) *	*p*-Value	CLDN-4 Low (*n* = 15) *	CLDN-4 High (*n* = 33) *	*p*-Value
Type of atrophy (%, *n*)			0.760			0.011
Closed type	17.6 (6)	21.4 (3)	0.760	40.0 (6)	9.1 (3)	0.011
Open type	82.4 (28)	78.6 (11)	0.760	60.0 (9)	90.9 (30)	0.011

CLDN, claudin. * Pre-operative endoscopy was performed in 48 patients.

## Data Availability

Not applicable.

## Supplementary Materials

The following supporting information can be downloaded at: https://www.mdpi.com/article/10.3390/jcm11123550/s1, Figure S1: Area under the curve of CLDN-4 expression for prediction of synchronous tumor.
