# Supplementary figures and images for: High Expression of Claudin-4 Is Associated with Synchronous Tumors in Patients with Early Gastric Cancer
Source: J Clin Med. 2022 Jun 20;11(12):3550. doi: 10.3390/jcm11123550 (PMC9224850; doi:10.3390/jcm11123550)

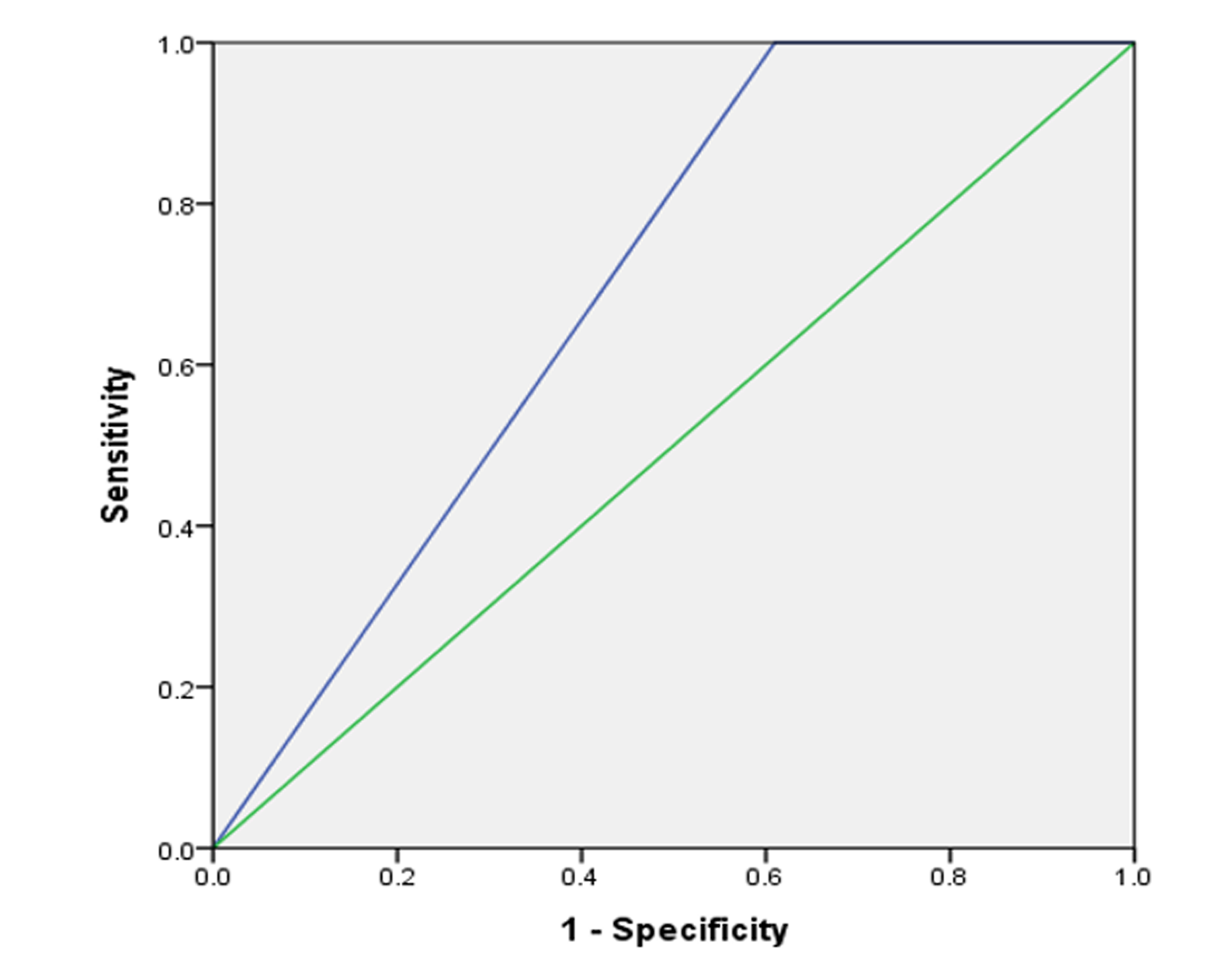

Supplement: Supplementary file 1 [file jcm-11-03550-s001.zip › jcm-1715824-supplementary.tif]
